# Epinephrine vs Norepinephrine as Initial Treatment in Children With Septic Shock

**DOI:** 10.1001/jamanetworkopen.2025.4720

**Published:** 2025-04-11

**Authors:** Matthew A. Eisenberg, Nathan Georgette, Alexandra H. Baker, Gregory P. Priebe, Michael C. Monuteaux

**Affiliations:** 1 Division of Emergency Medicine, Department of Pediatrics, Boston Children’s Hospital, Boston, Massachusetts; 2 Department of Pediatrics, Harvard Medical School, Boston, Massachusetts; 3 Department of Emergency Medicine, Harvard Medical School, Boston, Massachusetts; 4 Division of Critical Care Medicine, Department of Anesthesiology, Critical Care and Pain Medicine, Boston Children’s Hospital, Boston, Massachusetts; 5 Division of Infectious Diseases, Department of Pediatrics, Boston Children’s Hospital, Boston, Massachusetts; 6 Department of Anaesthesia, Harvard Medical School, Boston, Massachusetts

## Abstract

**Importance:**

There is no consensus and wide practice variation in the choice of initial vasoactive agent in children with septic shock.

**Objective:**

To determine whether receipt of epinephrine compared with norepinephrine as the first vasoactive medication administered is associated with improved outcomes among children with septic shock without known cardiac dysfunction.

**Design, Setting, and Participants:**

This single-center, retrospective cohort study used propensity score matching to examine encounters in which a patient was diagnosed with septic shock and required a vasoactive infusion within 24 hours of ED arrival at a freestanding quaternary care children’s hospital. Participants included patients aged 1 month to 18 years who presented to the ED and were diagnosed with septic shock without known cardiac dysfunction and began an epinephrine or norepinephrine infusion within 24 hours of ED arrival between June 1, 2017, and December 31, 2023. Data were analyzed from March 1 to December 31, 2024.

**Exposure:**

Epinephrine vs norepinephrine as the first vasoactive medication received.

**Main Outcomes and Measures:**

The primary outcome was major adverse kidney events by 30 days (MAKE30). Secondary outcomes were 30-day in-hospital mortality, 3-day mortality, need for kidney replacement therapy or persistent kidney dysfunction, endotracheal intubation, mechanical ventilation days, extracorporeal membrane oxygenation, and hospital and intensive care unit length of stay. Primary and secondary outcomes were assessed with the χ^2^ test of proportions for binary variables and Wilcoxon rank sum test for continuous variables.

**Results:**

Among 231 included encounters, the median (IQR) age was 11.4 (5.6-15.4) years, 126 were female (54.6%), and 142 had a medical history that predisposed them to sepsis (61.5%). Most (147 [63.6%]) initially received an epinephrine infusion and 84 (36.4%) received norepinephrine. In the epinephrine group, 9 of 147 (6.1%) met the outcome of MAKE30 and 6 of 147 (4.1%) died within 30 days. In the norepinephrine group, 3 of 84 (3.6%) met MAKE30 and there were no deaths. After inverse probability of treatment weighting, there were no significant differences in the primary outcome, MAKE30. With 2:1 propensity matching, epinephrine was associated with greater 30-day mortality compared with norepinephrine (3.7% vs 0%; risk difference: 3.7%; 95% CI, 0.2%-7.2%).

**Conclusions and Relevance:**

In this study, those receiving epinephrine had greater 30-day mortality but no difference in MAKE30. Prospective, confirmatory studies are needed to determine if norepinephrine should be the first-line vasoactive agent in pediatric septic shock.

## Key Points

**Question:**

Is there an association between the first vasoactive agent administered and outcomes in children with septic shock without known cardiac dysfunction?

**Findings:**

In this retrospective cohort study of 231 encounters using propensity matching, there was no difference in the primary outcome, major adverse kidney events by 30 days, between patients receiving epinephrine vs norepinephrine, but epinephrine was associated with greater 30-day mortality.

**Meaning:**

These findings suggest that outcomes were different for patients treated with epinephrine vs norepinephrine as the initial agent, supporting the need for prospective, confirmatory studies.

## Teaser

This cohort study assesses whether receiving epinephrine compared with norepinephrine as the first vasoactive medication administered is associated with improved outcomes for children with septic shock at a pediatric hospital.

## Introduction

Sepsis is a leading cause of morbidity and mortality in children worldwide.^1^ While quality improvement efforts^2,3,4^ and well-publicized practice guidelines^5,6,7^ have led to declining sepsis mortality in the US since 2004, between 4% and 10% of all US children with sepsis will die from their illness.^8,9,10,11^ The majority of sepsis-attributable deaths are due to refractory shock, or multiorgan dysfunction following shock recovery.^12^

For children with sepsis with persistent shock after appropriate fluid resuscitation, vasoactive agents are the treatment of choice to increase blood pressure and restore tissue perfusion.^6,7^ National guidelines previously recommended dopamine as the first-line vasoactive medication in the treatment of children with sepsis^5^ until 2 studies showed lower mortality and fewer organ failure days in children treated with epinephrine rather than dopamine.^13,14^ Revised guidelines subsequently recommended either epinephrine or norepinephrine as the first vasoactive medication administered, with the American College of Critical Care Medicine (ACCM) favoring epinephrine while the Surviving Sepsis Campaign guidelines were unable to issue a recommendation between the 2 due to insufficient evidence.^6,7^ This is in contrast to adult guidelines, which recommend norepinephrine as the first-line vasoactive medication in septic shock.^15^ To date, no studies have directly compared epinephrine with norepinephrine in the treatment of pediatric septic shock.

Norepinephrine and epinephrine both have vasopressor and inotropic effects,^16^ are inexpensive, and can be administered safely through either a peripheral intravenous catheter or a central venous line.^17,18^ Both have been shown to be safe and effective in increasing blood pressure and urine output in children,^19^ and both are effective in the treatment of adult septic shock.^20^ Determining whether either confers better outcomes in pediatric septic shock could benefit the hundreds of thousands of children diagnosed with sepsis annually.^9^

## Methods

### Study Design

This cohort study follows the Strengthening the Reporting of Observational Studies in Epidemiology (STROBE) reporting guideline.^24^ The Boston Children’s Hospital institutional review board approved the study with a waiver of informed consent. We performed a retrospective cohort study of encounters in which a patient was diagnosed with septic shock requiring a vasoactive infusion within 24 hours of emergency department (ED) arrival.

### Objective

To determine whether receipt of epinephrine compared with norepinephrine as the first vasoactive medication was associated with a decrease in major adverse kidney events by 30 days (MAKE30) in children with septic shock without known cardiac dysfunction. The secondary objective was to determine whether there was a difference in 30-day in-hospital mortality in this same population.

### Population

Encounters of patients aged 1 month to 18 years presenting to the ED of a freestanding, quaternary care pediatric hospital with septic shock within 24 hours of ED arrival between June 1, 2017, and December 31, 2023, were included. The hospital has approximately 70 000 annual ED visits and 450 inpatient beds. The start date was chosen because the ACCM guidelines advocating for epinephrine or norepinephrine as the first-line vasoactive medication in septic shock were released that month^7^; prior to that, dopamine was considered to be the first-line vasoactive medication for most children with septic shock.^5^

Encounters were included if the patient presented to the ED, received an IV antibiotic and was initiated on a norepinephrine or epinephrine infusion for septic shock (as reflected in the physician note in the electronic health record) within 24 hours of ED arrival. These criteria were chosen to select patients with community-acquired as opposed to hospital-acquired sepsis. Exclusion criteria were: (1) anaphylaxis, defined as administration of intramuscular epinephrine prior to the vasoactive infusion; (2) admission to the cardiac intensive care unit; (3) diagnosis of myocarditis; (4) patients receiving home medications for treatment of heart failure; (5) an echocardiogram indicating impaired function that was performed after ED arrival but prior to initiation of the vasoactive medicine; (6) cardiac arrest prior to initiation of the vasoactive medicine; (7) patients transferred into the ED already on a vasoactive infusion; and (8) documentation that the vasoactive medicine was initiated for a reason other than sepsis (eg, trauma or in response to adverse effects of sedation). Medical records of potentially eligible patients were manually reviewed by 1 of the study authors (M.E., N.G., or A.B.) for the presence of exclusion criteria.

### Variables and Outcomes

The primary variable was the first vasoactive medication administered—epinephrine or norepinephrine (with norepinephrine designated as the referent). Additional variables are shown in Table 1. Mechanical ventilation includes only invasive ventilation, not continuous or biphasic positive airway pressure. Race and ethnicity data were extracted from the electronic health record, which was based on self report of the patient or family at the time of hospital registration.

**Table 1.** Baseline Characteristics of Children With Septic Shock Within 24 Hours of ED Arrival According to First Vasoactive Medication Administered

Characteristics	Patients, No. (%)			*P* value
	All (n = 231)	Epinephrine (n = 147)	Norepinephrine (n = 84)	
Age, median (IQR), y	11.4 (5.6-15.4)	10.5 (5.2-14.8)	12.2 (5.9-16.0)	.05
Race				
Asian, non-Hispanic	12 (5.2)	8 (5.4)	4 (4.8)	.73
Black, non-Hispanic	23 (10.0)	17 (11.6)	6 (7.1)	
Hispanic	35 (15.2)	23 (15.7)	12 (14.3)	
White, non-Hispanic	112 (48.5)	66 (44.9)	46 (54.8)	
Another race	30 (13.0)	21 (14.3)	9 (10.7)	
Multiracial	5 (2.2)	4 (2.7)	1 (1.2)	
Unknown	14 (6.1)	8 (5.4)	6 (7.1)	
Sex				
Male				
Female	126 (54.6)	77 (52.4)	49 (58.3)	.38
Sepsis risk factor^a^	142 (61.5)	89 (60.5)	53 (63.1)	.70
Malignant neoplasm	44 (19.1)	24 (16.3)	20 (23.8)	.16
Asplenia	3 (1.3)	2 (1.4)	1 (1.2)	.91
Bone marrow transplant	10 (4.3)	3 (2.0)	7 (8.3)	.02
CVL or PICC	47 (20.4)	26 (17.7)	21 (25.0)	.18
Transplant	4 (1.7)	3 (2.0)	1 (1.2)	.63
Neurological impairment	71 (30.7)	47 (32.0)	24 (28.6)	.59
Other	18 (7.8)	13 (8.8)	5 (6.0)	.43
Blood culture positive^b^	37/203 (18.2)	25/132 (18.9)	12/71 (16.9)	.72
Fluid volume prior to vasoactive (IQR), mL/kg	49.8 (31.9-60.1)	44.1 (30.0-60.0)	50.3 (37.4-60.4)	.12
Tachycardia prior to vasoactive administration^c^	153 (66.2)	98 (66.7)	55 (65.5)	.85
Lactic acid, median, mmol/L^d^	1.9 (1.2-3.0)	2.1 (1.3-3.2)	1.6 (1.0-2.6)	.10
No. of observations	179	111	68	NA
Mechanical ventilation prior to vasoactive	46 (19.9)	28 (19.1)	18 (21.4)	.66
Etomidate prior to vasoactive	19 (8.2)	13 (8.8)	6 (7.1)	.65
Time from arrival to vasoactive, median (IQR), h	4.7 (2.6-8.5)	3.6 (2.2-6.1)	7.5 (4.6-13.1)	<.001
ED start of vasoactive	144 (62.3)	115 (78.2)	29 (34.5)	<.001
Maximum Phoenix score prior to vasoactive, median (IQR)	1 (0.5-3.0)	1 (0-3)	1 (1-2)	NA
Total cardiovascular	1 (0-1)	1 (0-1)	1 (0-1)	.79
Lactate score	0	0	0	
MAP score	1 (0-1)	1 (0-1)	0.5 (0-1)	
Respiratory	0 (0-1)	0 (0-1)	0 (0-1)	
Coagulation	0 (0-1)	0 (0-1)	0 (0-1)	
Neurologic	0	0	0	

Abbreviations: CVL, central venous line; ED, emergency department; MAP, mean arterial pressure; NA, not applicable; PICC, peripherally inserted central catheter.

^a^ Patients could have more than 1 sepsis risk factor.

^b^ Positive blood culture defined as growth of bacteria on first blood culture obtained on day of ED arrival.

^c^ Proportion with last recorded heart rate prior to initiation of the vasoactive infusion higher than upper limit for age by pediatric advanced life support.

^d^ Lactic acid reported is last value prior to initiation of the vasoactive agent.

The primary outcome was MAKE30, a composite outcome of death, new kidney replacement therapy or persistent kidney dysfunction by 30 days or hospital discharge, whichever came first.^21^ Secondary outcomes were 30-day in-hospital mortality, 3-day in-hospital mortality, receipt of extracorporeal membrane oxygenation (ECMO), endotracheal intubation following initiation of the vasoactive infusion, persistent kidney dysfunction or need for new kidney replacement therapy (defined in the same way as MAKE30), receipt of additional vasoactive agents, receipt of steroids (defined as first dose of IV hydrocortisone given after vasoactive agent start time), mechanical ventilation-free days (out of 30 days), intensive care unit (ICU)-free days (out of 30 days) and hospital-free days (out of 30 days). For the latter 3 measures, patients who died had mechanical ventilation-free days, hospital-free days and ICU-free days set to 0. Finally, we assessed for the presence of tachyarrhythmias, a known potential adverse effect of vasoactive therapy, by querying the record for administration of any of the following antiarrhythmic medications between vasoactive start time and hospital discharge: adenosine, amiodarone, diltiazem, flecainide, lidocaine, procainamide, propranolol, or sotalol.

### Statistical Analysis

Demographic and hospital course data were summarized using frequencies and percentages for categorical variables and medians and IQR for continuous variables. Primary and secondary outcomes were assessed with the χ^2^ test of proportions for binary variables and Wilcoxon rank sum test for continuous variables.

#### Inverse Probability Weighting

To account for differences in baseline characteristics between patients who received epinephrine vs norepinephrine, we conducted a propensity score analysis using inverse probability of treatment weighting (IPTW). This method was chosen in order to account for confounding but allow all eligible subjects to be included in the analysis. We used the following characteristics to generate the propensity scores, chosen a priori based on known or suspected association with the outcomes: age, presence of a sepsis risk factor, time from ED arrival to first vasoactive infusion, presence of a positive blood culture, amount of fluid administered prior to vasoactive infusion, and highest Phoenix Sepsis Organ Dysfunction Score^22^ total prior to initiation of the vasoactive agent. Blood cultures were considered positive if the first blood culture sent for the encounter grew a bacterial or fungal pathogen. We used standardized differences to assess the balance of covariates between children who received epinephrine vs norepinephrine as their first vasoactive medication. The results of the IPTW analysis provide the average treatment outcome: the risk of each outcome if all patients were treated with epinephrine compared with the baseline risk that would be observed if all patients were treated with norepinephrine. Covariates with standardized differences less than 0.1 were considered balanced.^23^ We also performed the overidentification test for covariate balance.

#### Secondary Analysis

Because no patients in the norepinephrine group experienced the secondary outcomes of 30-day in-hospital mortality, 3-day in-hospital mortality, or ECMO, the IPTW analysis was not feasible for these outcomes. Therefore, we made the post hoc decision to conduct a secondary analysis using a propensity score matching approach, which allowed for the estimation of the comparison. We conducted nearest neighbor matching where each patient was matched with at least two patients from the other treatment level. We set the caliper (ie, the maximum allowable difference between propensity scores of matched patients) at 0.2 times the standard deviation of the logit of the propensity score.^23^ The covariates were the same variables used in the primary analysis.

Data processing was performed with Python version 3.11 (Python Software Foundation) and statistical analyses were completed using Stata 18 (StataCorp). Where applicable, *P* < .05 were considered statistically significant. All tests were 2-sided. Data were analyzed from March 1 to December 31, 2024.

## Results

### Characteristics

During the study period, there were 434 encounters that met the inclusion criteria, from which 231 were included in the analysis (Figure). The median (IQR) age among participants was 11.4 (5.6-15.4) years, 126 (54.6%) were female sex and 142 (61.5%) had a previous medical history that predisposed them to sepsis. Most patients (147 of 231 [63.6%]) initially received an epinephrine infusion while 84 of 231 (36.4%) received norepinephrine first. Baseline differences between these 2 groups are shown in Table 1. Only time to initiation of vasoactive infusion and location where the vasoactive medication was started (ED vs ICU) were significantly different between the 2 groups.

**Figure.**
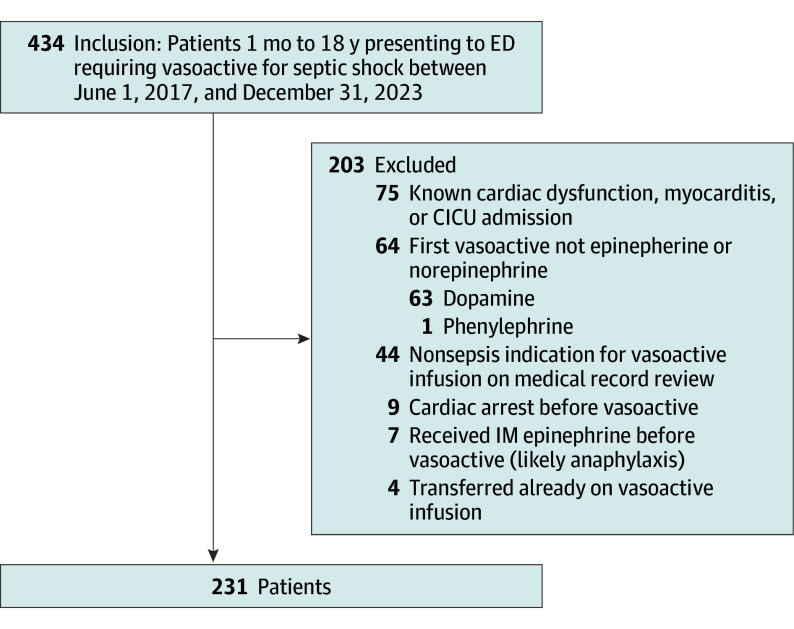
Patient Selection Flowsheet CICU indicates cardiac intensive care unit; ED, emergency department; IM, intramuscular.

### Main Results

Overall, 12 patients (5.2%) met the primary outcome of MAKE30; 9 of 147 (6.1%) in the epinephrine group and 3 of 84 patients (3.6%) in the norepinephrine group. There were 6 deaths within 30 days of ED arrival (2.6%)—all in the epinephrine group, which had a 30-day in-hospital mortality rate of 4.1% (Table 2).

**Table 2.** Unadjusted Outcomes by First Vasoactive Medication Given^a^

Outcome	All (n = 231)	Epinephrine (n = 147)	Norepinephrine (n = 84)	Risk difference (95% CI), %
MAKE30	12 (5.2)	9 (6.1)	3 (3.6)	2.6 (−3.0 to 8.1)
Death within 30 d	6 (2.6)	6 (4.1)	0	4.1 (0.9 to 7.3)
Death within 3 d	5 (2.2)	5 (3.4)	0	3.4 (0.5 to 6.3)
Need for new KRT	2/228 (0.9)	1/145 (0.7)	1/83 (1.2)	−0.5 (−3.2 to 2.2)
Persistent renal dysfunction	8/228 (3.5)	6/145 (4.1)	2/83 (2.4)	1.7 (−2.9 to 6.4)
Intubation after vasoactive	33/185 (17.8)	26/119 (21.9)	7/66 (10.6)	11.2 (−0.7 to 21.7)
Mechanical ventilation free days at 30 d, mean (SD), d	26.4 (9.0)	25.8 (9.7)	27.4 (7.7)	−1.6 (−4.0 to 0.9)^b^
ICU free days at 30 d, mean (SD), d	20.5 (10.3)	19.9 (10.7)	21.5 (9.5)	−1.7 (−4.4 to 1.1)^b^
Hospital free days at 30 d, mean (SD), d	16.5 (10.5)	16.4 (10.7)	16.8 (10.2)	−0.4 (−3.3 to 2.4)
ECMO	1/230 (0.4)	1/146 (0.7)	0/84 (0.0)	0.7 (−0.7 to 2.0)
First dose of hydrocortisone after vasoactive	57/212 (26.9)	41/138 (29.7)	16/74 (21.6)	8.1 (−4.0 to 20.2)
No. of additional vasoactives				
0	146 (63.2)	77 (52.4)	69 (82.1)	29.8 (18.3 to 41.3)^c^
1	67 (29.0)	55 (37.4)	12 (14.3)	
2	14 (6.1)	12 (8.2)	2 (2.4)	
>2	4 (1.7)	3 (2.0)	1 (1.2)	

Abbreviations: ECMO, extracorporeal membrane oxygenation; ICU, intensive care unit; KRT, kidney replacement therapy; MAKE30, major adverse kidney events at 30 days.

^a^ For binary outcomes the No. and percentage are reported. For continuous outcomes the mean and standard deviation are reported.

^b^ Difference in means by 2-sample *t* test.

^c^ Difference in proportion for more than 1 additional vasoactive vs no additional vasoactive agents.

For the IPTW analysis, adequate balance between treatment groups on the covariates was found, as indicated by a standardized difference less than 0.1 for each covariate in the model (Table 3). We did not detect compelling evidence that the overlap assumption was violated (eFigure in Supplement 1). Table 4 shows the results of the IPTW analysis. There was not a statistically significant difference in the primary outcome, MAKE30, nor in any of the secondary outcomes with the exception of administration of additional vasoactive medications, which was more common in the epinephrine group. Among those in the epinephrine group, 70 patients (47.6%) received at least 1 additional vasoactive (norepinephrine, 66 [44.9%]; vasopressin, 10 [6.8%]; dopamine, 9 [6.1%]; phenylephrine, 2 [1.4%]). In the norepinephrine group 15 patients (17.9%) received at least 1 additional vasoactive: (epinephrine, 14 [16.7%]; vasopressin, 3 [3.6%]; dopamine, 1 (1.2%); and phenylephrine, 1 [1.2%]). No patients in either group experienced a tachyarrhythmia requiring medication. We were unable to evaluate the 3-day and 30-day mortality and ECMO outcomes using IPTW because the model failed to estimate the treatment effect due to spare data (ie, none of the patients in the norepinephrine group experienced these outcomes).

**Table 3.** Standard Differences in Variables Before and After IPTW^**a**^

Variable	Results before weighting		Standardized difference^b^	
	Epinephrine (n = 147)	Norepinephrine (n = 84)	Preweighting	Postweighting
Phoenix score prior to vasoactive, mean (SD)	1.77 (1.73)	1.79 (1.55)	−0.010	0.003
Age, mean (SD), y	10.02 (5.41)	11.12 (5.60)	−0.198	−0.001
Any sepsis risk factor, proportion (SD)	0.61 (0.49)	0.63 (0.49)	−0.052	−0.009
Fluid volume prior to vasoactive, mean (SD), mL/kg	45.51 (22.53)	50.21 (23.30)	−0.205	−0.078
Blood culture positive, proportion (SD)	0.17 (0.37)	0.14 (0.35)	0.075	−0.033
Time from arrival to vasoactive, mean (SD), h	4.89 (4.10)	9.21 (6.04)	−0.837	0.006
Female sex, proportion (SD)	0.52 (0.50)	0.58 (0.50)	−0.119	−0.088
Tachycardia prior to vasoactive administration, proportion (SD)^b^	0.67 (0.22)	0.65 (0.23)	−0.025	0.011
Lactic acid, mean (SD), mmol/L	2.63 (2.00)	2.30 (2.06)	0.162	−0.110
Mechanical ventilation prior to vasoactive, proportion (SD)	0.19 (0.40)	0.21 (0.41)	−0.059	0.163
Etomidate prior to vasoactive, proportion (SD)	0.09 (0.28)	0.07 (0.26)	0.62	0.052
ED start of vasoactive, proportion (SD)	0.78 (0.41)	0.35 (0.48)	0.977	0.453

Abbreviations: ED, emergency department; IPTW, inverse probability of treatment weighting.

^a^ The IPTW and propensity score matching models included the Phoenix Sepsis Organ Dysfunction Score, age, risk factor presence, bolus amount before vasoactive, first blood culture positivity, and hours to vasoactive initiation.

^b^ Standardized difference reported is the standardized mean difference for all continuous variables and the standardized risk difference for all proportions.

**Table 4.** Inverse Probability of Treatment–Weighted Outcomes for Patients With Septic Shock by Initial Vasoactive Medication Administered

Outcome	Risk difference (95% CI), %		
	Epinephrine	Norepinephrine	Average treatment effect of epinephrine^a^
MAKE30	5.4 (1.9 to 8.9)	3.3 (−0.6 to 7.1)	2.1 (−3.2 to 7.3)
KRT or persistent kidney dysfunction (n = 228)	3.5 (0.7 to 6.4)	3.4 (−0.6 to 7.4)	0.1 (−4.8 to 5.0)
Intubation after vasoactive (n = 185)	22.4 (14.0 to 30.7)	13.9 (5.3 to 22.5)	8.5 (−2.3 to 19.2)
Mechanical ventilation free days at 30 d	25.8 (24.1 to 27.4)	26.6 (24.0 to 29.2)	−0.85 (−3.8 to 2.1)
ICU free days at 30 d	19.2 (17.4 to 21.1)	20.8 (18.5 to 23.1)	−1.6 (−4.2 to 1.0)
Hospital free days at 30 d	15.5 (13.6 to 17.4)	16.3 (13.9 to 18.7)	−0.8 (−3.5 to 1.9)
First dose of hydrocortisone after vasoactive	27.2 (19.4 to 35.0)	22.8 (12.1 to 33.4)	4.4 (−8.5 to 17.4)
Additional vasoactive	45.9 (37.0 to 54.9)	21.3 (10.7 to 31.8)	24.6 (11.6 to 37.7)

Abbreviations: ICU, intensive care unit; KRT, kidney replacement therapy; MAKE30, major advanced kidney events at 30 days.

^a^ The average treatment effect refers to that of epinephrine vs that of norepinephrine. 95% CI presented in parentheses.

### Secondary Analysis

In the secondary analysis using a propensity matching approach, all covariates were again well balanced between treatment groups as indicated by standard differences less than 0.1 (eTable in Supplement 1). All patients were included in the analysis as all propensity scores were within the prespecified caliper used to establish matches. Epinephrine was associated with greater 30-day mortality (3.7 vs 0%; risk difference: 3.7%; 95% CI, 0.2% to 7.2%), but not 3-day mortality (3.2% vs 0%; risk difference 3.2%; 95% CI, −0.2% to 6.7%) or use of extracorporeal membrane oxygenation (0.4% vs 0%; risk difference 0.4%; 95% CI, −0.4% to 1.3%).

## Discussion

In this propensity-matched cohort of children with sepsis without known cardiac dysfunction requiring a vasoactive infusion, we found that use of norepinephrine was associated with similar rates of MAKE30 but lower mortality compared with epinephrine. MAKE30 is a composite outcome with 2 components: mortality and need for kidney replacement therapy or persistent renal dysfunction at day 30 or hospital discharge. Our findings suggest that norepinephrine was associated with a benefit in terms of the mortality component, but not the renal component, of MAKE30.

To our knowledge, this is the first study to directly compare norepinephrine with epinephrine in the treatment of pediatric septic shock. A recent meta-analysis^25^ of vasoactive medications in this population suggested a mortality benefit for norepinephrine compared with epinephrine or dopamine, but the 95% CI overlapped substantially. On the other hand, our study showed a 30-day mortality benefit for norepinephrine. If prospectively confirmed, routine use of norepinephrine as the first-line agent for pediatric septic shock without known cardiac dysfunction could confer a mortality benefit in pediatric sepsis.

The 2020 Surviving Sepsis guidelines noted that “evidence is insufficient to recommend either epinephrine or norepinephrine as the initial vasoactive agent for children with fluid-resistant septic shock.”^6^ This absence of clarity has led to wide practice variation. In a survey of Surviving Sepsis Campaign panelists, they found that both medications were used in equal numbers, with a general preference toward using norepinephrine in patients with low systemic vascular resistance and epinephrine in children with myocardial dysfunction or other low cardiac-output states.^6^ Similarly, in a survey of European intensivists given the same clinical vignette of a child in septic shock, 60% chose norepinephrine as their vasoactive agent of choice, 25% epinephrine, with the rest choosing dobutamine, dopamine or a combination of the aforementioned.^26^ This practice variation suggests that standardized guidance, based on high quality evidence, may help improve pediatric sepsis outcomes.

It is not clear from our study the mechanism through which norepinephrine may have been associated with decreased mortality. Norepinephrine and epinephrine both stimulate the peripheral α-1 adrenergic receptors, while epinephrine has stronger affinity for the β-1 and β-2 receptors.^27,28^ As a result, norepinephrine is a more potent vasoconstrictor while epinephrine is a stronger inotropic agent. Norepinephrine is also known to increase preload, which helps to increase cardiac output. Epinephrine has been shown to cause higher rates of metabolic and cardiac complications in septic adults, particularly tachydysrhythmias,^29,30^ although in our study no patients in either group developed a tachyarrhythmia requiring medication. While these findings were thought to be less applicable to children without underlying cardiovascular disease, it is notable that an animal model of sepsis involving young, healthy dogs (ie, aged 12 to 28-month) likewise found that epinephrine adversely affected organ function, systemic perfusion, and survival compared with norepinephrine and vasopressin.^31^

Notably, improved mortality with norepinephrine was not mediated by decreased kidney injury in this study, as there was no difference in rates of new kidney replacement therapy or persistent kidney dysfunction between the 2 groups. Further study will be necessary to determine the mechanism through which norepinephrine was associated with a survival benefit in this population.

Alternatively, while we propensity matched based on patient characteristics, comorbidities, and measures of organ dysfunction, our results may still represent unmeasured confounding. Clinicians may have chosen epinephrine specifically for patients with known or suspected cardiac dysfunction,^32^ and these patients might be expected to have worse outcomes. For this reason, we excluded patients with heart failure, cardiac dysfunction on echocardiogram prior to the time of vasoactive medication initiation, and those admitted to the cardiac ICU, but we were unable to exclude patients with suspected cardiac dysfunction based on examination findings or point of care ultrasonography alone. Additionally, patients in the epinephrine group had their vasoactive medication started sooner after ED arrival than those in the norepinephrine group, raising the possibility that patients receiving epinephrine were sicker than patients receiving norepinephrine. Although in the propensity matched samples the time to vasoactive initiation was similar, as was the Phoenix organ dysfunction score prior to vasoactive start time, there may have been other markers of illness severity that we were not able to account for. The greater frequency of additional vasoactive agents in the epinephrine group, including crossover to norepinephrine, likewise may reflect sicker patients in the epinephrine group or, alternatively, that norepinephrine alone was more effective at treating shock. Only a prospective study can discern whether these differences are due to disease severity or treatment effect.

### Limitations

This study has limitations. First, this was a single-center study, and our results may not be generalizable to patients with septic shock seen at other institutions. Additionally, our sample size may not have allowed us to detect rare outcomes or to determine whether subsets of sepsis patients (eg, those with malignant neoplasm) had different results from the study population as a whole. Third, although we used propensity matching to adjust for known confounders and degree of organ dysfunction (as measured by the Phoenix sepsis score), unmeasured confounders, as noted previously, may have affected the results, particularly unmeasured patient characteristics that led the clinician to choose epinephrine or norepinephrine that were also risk factors for MAKE30 or death. It is notable that most patients in the epinephrine group had their vasoactive medication started in the ED, while most in the norepinephrine group had it started in the intensive care unit. It is not clear what association this may have had with outcomes, especially after matching on time to initiation of the infusion and organ dysfunction score.

For patients who were transferred into our hospital after first receiving therapy at an outside institution, we were unable to precisely determine time to first antibiotic, fluid volumes, and time to initiation of vasoactive medications, though this is unlikely to bias the results in any particular direction. Additionally, we were unable to ascertain the vasoactive medication doses or duration of therapy, though in practice most clinicians likely used standardized dose ranges titrated to blood pressure parameters. Finally, we only analyzed patients according to the first vasoactive medication received, although many patients received multiple vasoactive medications during their hospital stay.

## Conclusions

These findings suggest that children without known cardiac dysfunction who received norepinephrine as the initial vasoactive agent for septic shock had lower mortality but similar rates of MAKE30 compared with children receiving epinephrine. Prospective, confirmatory studies are needed to determine whether norepinephrine should be the first vasoactive agent given to children with septic shock without known cardiac dysfunction.

## Notes

Supplement 1. **eTable.** Characteristics of Children With Septic Shock by Initial Vasoactive Medication Administered Following Propensity Score Matching **eFigure.** Distribution of Propensity Scores for Patients in Epinephrine (Red) vs Norepinephrine (Blue) Groups

Supplement 2.
Data Sharing Statement
