# Supplementary material for: Epinephrine vs Norepinephrine as Initial Treatment in Children With Septic Shock
Source: JAMA Netw Open. 2025 Apr 11;8(4):e254720. doi: 10.1001/jamanetworkopen.2025.4720 (PMC11992602; doi:10.1001/jamanetworkopen.2025.4720)
Supplement: Supplement 1. — eTable. Characteristics of Children With Septic Shock by Initial Vasoactive Medication Administered Following Propensity Score Matching eFigure. Distribution of Propensity Scores for Patients in Epinephrine (Red) vs Norepinephrine (Blue) Groups [file jamanetwopen-e254720-s001.pdf]

Supplemental Online Content

Eisenberg MA, Georgette N, Baker AH, Priebe GP, Monuteaux MC. Epinephrine vs norepinephrine as initial treatment in children with septic shock. *JAMA Netw Open*. 2025;8(5):e254720. doi:10.1001/jamanetworkopen.2025.4720

eTable. Characteristics of Children With Septic Shock by Initial Vasoactive Medication Administered Following Propensity Score Matching

eFigure. Distribution of Propensity Scores for Patients in Epinephrine (Red) vs Norepinephrine (Blue) Groups

This supplemental material has been provided by the authors to give readers additional information about their work.

© 2025 Eisenberg MA et al. *JAMA Network Open*.

eTable. Characteristics of Children With Septic Shock by Initial Vasoactive Medication Administered Following Propensity Score Matching

	**After Propensity Score Matching**
	**Absolute Standard Difference[#]**
PSS	0.051
Age	0.054
Risk factor presence	0.079
Bolus amount prior to vasoactive (mL/kg)	-0.014
First blood culture positivity	0.030
Hours to vasoactive initiation	-0.024

[#]Absolute standard difference reported is the standardized mean difference for all continuous variables and the risk difference for all proportions after propensity matching with 2 nearest neighbors and caliper set at 0.2 times the standard deviation of the logit of the propensity score.

eFigure. Distribution of propensity scores for patients in epinephrine (red) vs. norepinephrine (blue) groups

© 2025 Eisenberg MA et al. *JAMA Network Open*.

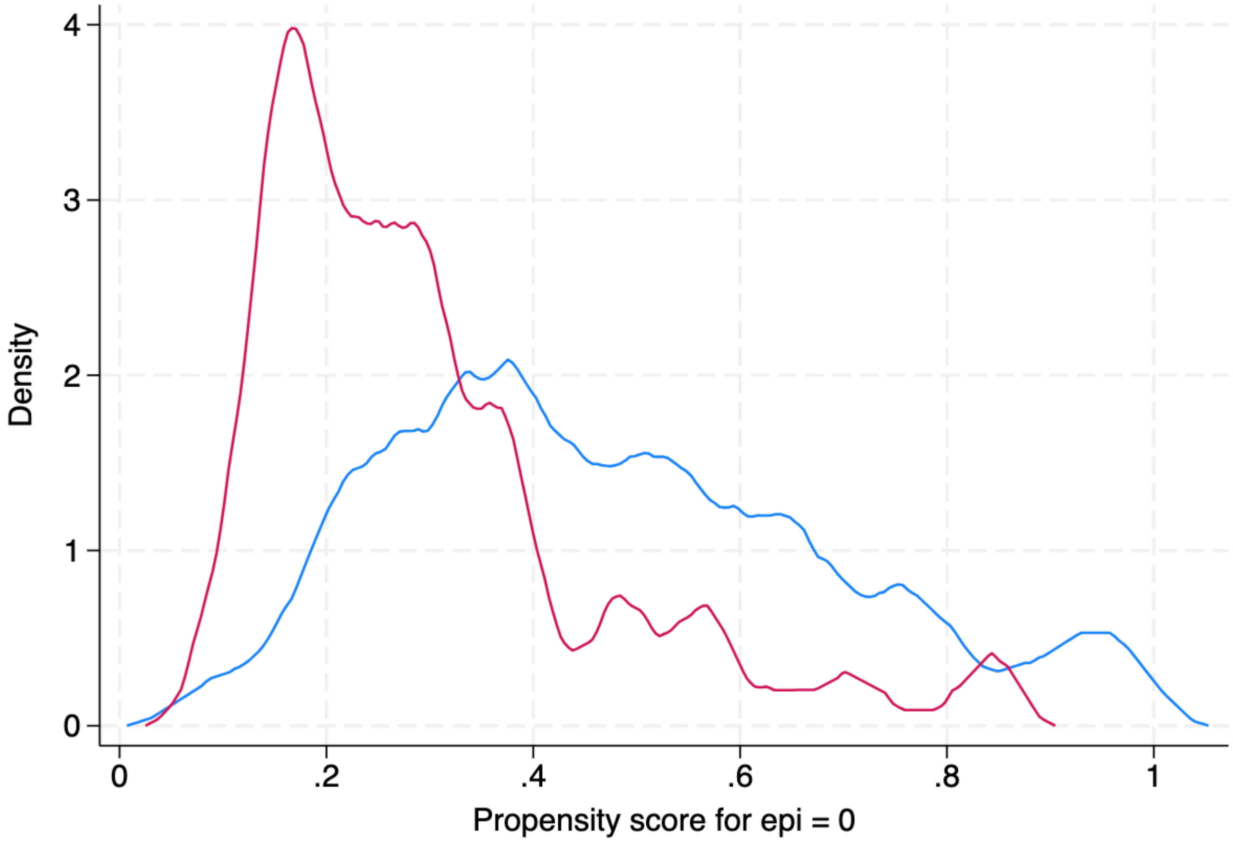

© 2025 Eisenberg MA et al. *JAMA Network Open*.
