# Supplementary material for: Epinephrine vs Norepinephrine as Initial Treatment in Children With Septic Shock
Source: JAMA Netw Open. 2025 Apr 11;8(4):e254720. doi: 10.1001/jamanetworkopen.2025.4720 (PMC11992602; doi:10.1001/jamanetworkopen.2025.4720)
Supplement: Supplement 2. — Data Sharing Statement [file jamanetwopen-e254720-s002.pdf]

Data Sharing Statement

Eisenberg. Epinephrine vs Norepinephrine as Initial Treatment in Children With Septic Shock. *JAMA Netw Open*. Published April 11, 2025. doi:10.1001/jamanetworkopen.2025.4720

Data
Data available: No

Additional Information
Explanation for why data not available: If necessary, can make deidentified data available after IRB review
